# Differential associations of childhood adversity subtypes and psychopathology in men and women

**DOI:** 10.1192/j.eurpsy.2023.254

**Published:** 2023-07-19

**Authors:** T. Prachason, I. Mutlu, L. Fusar-Poli, C. Menne-Lothmann, J. Decoster, R. van Winkel, D. Collip, P. Delespaul, M. De Hert, C. Derom, E. Thiery, N. Jacobs, M. Wichers, J. van Os, B. P. F. Rutten, L.-K. Pries, S. Gülöksüz

**Affiliations:** 1 Department of Psychiatry and Neuropsychology, School for Mental Health and Neuroscience, Maastricht University Medical Center, Maastricht, Netherlands; 2 Institute of Graduate Programs, Department of Clinical Psychology, Istanbul Bilgi University, Istanbul, Türkiye; 3 Department of Brain and Behavioral Sciences, University of Pavia, Pavia, Italy; 4 Department of Neurosciences, University Psychiatric Centre KU Leuven, KU Leuven; 5 Brothers of Charity, University Psychiatric Centre Sint-Kamillus Bierbeek, Bierbeek; 6 University Psychiatric Centre Katholieke Universiteit Leuven, Kortenberg; 7 Department of Neurosciences, Centre for Clinical Psychiatry, Katholieke Universiteit Leuven; 8 Leuven Brain Institute, Katholieke Universiteit Leuven, Leuven; 9 Antwerp Health Law and Ethics Chair, University of Antwerp, Antwerp; 10 Centre of Human Genetics, University Hospitals Leuven, KU Leuven; 11 Department of Obstetrics and Gynecology, Ghent University Hospitals; 12 Department of Neurology, Ghent University Hospital, Ghent University, Ghent, Belgium; 13 Faculty of Psychology, Open University of the Netherlands, Heerlen; 14 Department of Psychiatry, Interdisciplinary Center Psychopathology and Emotion Regulation (ICPE), University of Groningen, University Medical Center Groningen, Groningen; 15 Department of Psychiatry, Brain Centre Rudolf Magnus, University Medical Centre Utrecht, Utrecht, Netherlands; 16 Department of Psychosis Studies, Institute of Psychiatry, King’s Health Partners, King’s College London, London, United Kingdom; 17 Department of Psychiatry, Yale School of Medicine, CT, United States

## Abstract

**Introduction:**

Prior evidence suggests that men and women might be differentially susceptible to distinct types of childhood adversity (CA), but research on gender-specific associations between CA subtypes and psychiatric symptoms is limited.

**Objectives:**

To test the gender-specific associations of CA subtypes and psychiatric symptoms in the general population.

**Methods:**

Data from 791 twins and siblings from the TwinssCan project were used. Psychopathology and CA exposure were assessed using the Symptom Checklist-90 Revised (SCL-90) and the Childhood Trauma Questionnaire (CTQ), respectively. The associations between the total CTQ scores and SCL-90 scores (i.e. total SCL-90, psychoticism, paranoid ideation, anxiety, depression, somatization, obsessive-compulsive, interpersonal sensitivity, hostility, and phobic anxiety) were tested in men and women separately. The associations between the five CA subtypes (i.e. physical abuse, emotional abuse, sexual abuse, physical neglect, and emotional neglect) and total SCL-90 were tested in a mutually adjusted model. As exploratory analyses, the associations between all CA subtypes and the nine SCL-90 subdomain scores were similarly tested. The regression coefficients between men and women were compared using Chow’s test. All models were adjusted for age and family structure.

**Results:**

Total CTQ was significantly associated with total SCL-90 in men (*B* = 0.013, *SE* = 0.003, *P* < .001) and women (*B* = 0.011, *SE* = 0.002, *P* < .001). The associations with the nine symptom domains were also significant in both genders (*P* < .001). No significant gender differences in the regression coefficients of total CTQ were detected. The analyses of CA subtypes showed a significant association between emotional abuse and total SCL-90 in women (*B* = 0.173, *SE* = 0.030, *P* < .001) and men (*B* = 0.080, *SE* = 0.035, *P* = .023), but the association was significantly stronger in women (ꭓ^2^(1) = 4.10, *P* = .043). The association of sexual abuse and total SCL-90 was only significant in women (*B* = 0.217, *SE* = 0.053, *P* < .001). The associations of emotional neglect (*B* = 0.061, *SE* = 0.027, *P* = .026) and physical neglect (*B* = 0.167, *SE* = 0.043, *P* < .001) with total SCL-90 were only significant in men. The explorative analyses of SCL-90 subdomains revealed significant associations of emotional abuse with all nine symptom domains and of sexual abuse with seven symptom domains in women. Significant associations of physical neglect with six symptom domains and of emotional neglect with depression were also detected in men. No other significant associations between CT subtypes and total SCL-90 or symptom domain scores were observed in men and women.

**Conclusions:**

CA exposure was associated with diverse psychopathology similarly in both genders. However, women are more sensitive to abuse, but men are more sensitive to neglect. Gender-specific influences of CA subtypes on psychopathology should be considered in future studies.

**Disclosure of Interest:**

None Declared

